# Group Sparse Representation Based on Nonlocal Spatial and Local Spectral Similarity for Hyperspectral Imagery Classification

**DOI:** 10.3390/s18061695

**Published:** 2018-05-24

**Authors:** Haoyang Yu, Lianru Gao, Wenzhi Liao, Bing Zhang

**Affiliations:** 1 Key Laboratory of Digital Earth Science, Institute of Remote Sensing and Digital Earth, Chinese Academy of Sciences, Beijing 100094, China; yuhy@radi.ac.cn (H.Y.); zb@radi.ac.cn (B.Z.); 2 College of Resources and Environment, University of Chinese Academy of Sciences, Beijing 100049, China; 3 Department of Telecommunications and Information Processing, IMEC-TELIN-Ghent University, 9000 Ghent, Belgium; wliao@telin.ugent.be

**Keywords:** hyperspectral imagery classification, group sparse representation (GSR), nonlocal spatial similarity, local spectral similarity

## Abstract

Spectral-spatial classification has been widely applied for remote sensing applications, especially for hyperspectral imagery. Traditional methods mainly focus on local spatial similarity and neglect nonlocal spatial similarity. Recently, nonlocal self-similarity (NLSS) has gradually gained support since it can be used to support spatial coherence tasks. However, these methods are biased towards the direct use of spatial information as a whole, while discriminative spectral information is not well exploited. In this paper, we propose a novel method to couple both nonlocal spatial and local spectral similarity together in a single framework. In particular, the proposed approach exploits nonlocal spatial similarities by searching non-overlapped patches, whereas spectral similarity is analyzed locally within the locally discovered patches. By fusion of nonlocal and local information, we then apply group sparse representation (GSR) for classification based on a group structured prior. Experimental results on three real hyperspectral data sets demonstrate the efficiency of the proposed approach, and the improvements are significant over the methods that consider either nonlocal or local similarity.

## 1. Introduction

Recent advances in remote sensing sensors, especially for hyperspectral imagery (HSI), increase the possibility of more accurate discrimination of materials of interest [1,2]. Given a set of observations (i.e., pixel vectors in a HSI), the purpose of classification is to assign a unique label to each pixel vector, such that it can be presented by a given class [3]. Although HSI is characterized by its high spectral resolution and abundant information, which promotes capturing fine details of spectral features for classification, it has been demonstrated that the original HSI contains high redundancy, and in addition there are high correlations in both the spectral and the spatial domains [4,5,6]. Therefore, the analysis problem is essentially low-rank and can be represented sparsely [7]. In this context, sparse representation (SR) has been widely exploited for HSI. SR-based classifiers (SRC) code a testing pixel over a dictionary which is constructed by sets of labeled samples [8,9]. The spectral features of pixels belonging to the same class are assumed to approximately lie in a lower-dimensional subspace and yield a relatively concentrated response under specific constraints towards the dictionary, such that the result is determined by the class with the minimum residual error of representation [10,11]. According to the constraints imposed on the coefficients, the representation-based classification can be divided into an *l*_2_-norm regularized collaborative representation-based classifier (CRC) and an *l*_1_-norm regularized SRC [12,13]. Several approaches based on SR for HSI have proved that it can provide plausible results compared with the traditional methods [14,15]. In [16], SR is imposed to the HSI, and was proven to be effective for the classification purpose. In [17], SRC is integrated with manifold learning-based dimensionality reduction methods, and provided considerable results for the classification of HSI. In [18], SR is fused with CR in a single classification framework based on a weighting process, and was proven to be better than both of them. Though SRC has shown its effectiveness for HSI classification, it only focuses on the usage of spectral information, and it ignores the inherent spatial information which would allow for more accurate discrimination [19,20].

In order to incorporate the spatial information, several models have been constructed for SR-based classification, such as imposing a smoothing constraint to the formulation or adding post processing in the framework [21,22,23]. Among these, Joint SRC (JSRC) has been of great interest due to its representation of pixels in a small neighborhood together, which are weighted by a different set of coefficients, and proven to be effective for HSI classification [24,25]. Though JSRC brings considerable improvement to SRC, SR-based approaches still suffer from instability of sparse coefficients due to the coherency in the dictionary. To address the problem, group SRC (GSRC) has been designed to exploit the spatial coherence and the inherent structure of dictionary based on group sparsity priors, such that a testing pixel with its neighborhood can be sparsely represented together with activation of group atoms instead of individual ones in the dictionary [26,27]. In [28], GSRC is firstly introduced for HSI classification, and proven to be reliable with a further development of a low rank group prior. In [29,30], structure sparsity priors are incorporated with manifold learning and subspace projection for better characterization, and achieved considerable results for classification. In comparison to SRC, GSRC considers spatial information through local area. However, it neglects nonlocal spatial information, where global spatial consistency can be further exploited for more comprehensive representation.

Compared with local similarity, nonlocal self-similarity (NLSS) defines the spatial consistency of materials in a global distribution [31]. It can provide references of global structure prior by exploiting the spatial similarity in nonlocal area, such that the discrimination of a pixel can be more precisely processed through the global similarity constraint [32]. Recently, several NLSS-based approaches have been designed for application to HSI [33,34]. In [35], a nonlocal means (NL-means) algorithm based on NLSS was proposed for HSI denoising, which estimates the value of pixels with weighted average of similar ones, and provides better results compared with other local smoothing filters. In [36], NL-means was applied as a regularization to exploit the similar structures in the abundance image, and proven to be effective by incorporating nonlocal spatial information for spectral unmixing. In [37], NLSS was introduced into SR to reconstruct the dictionary for separation of signal and noise, and contributed to more concise and accurate restoration of HSI. Though these NLSS-based methods have shown their superiority based on global structured priors in spectral and spatial domains, they essentially tend to the direct use of nonlocal spatial similarity with concentration of spatial information from all the available regions, while the spectral features in fact can still be exploited for more accurate discrimination.

**Figure 1:**
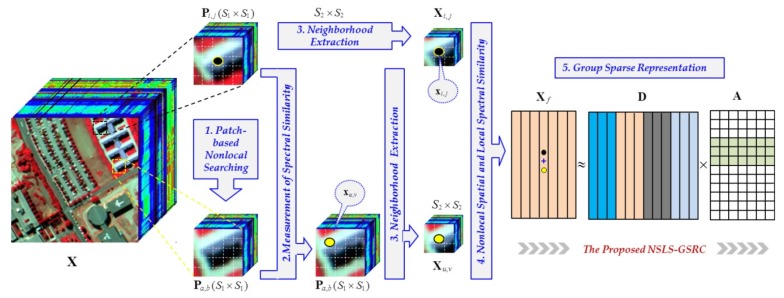
General framework. (X: a HSI; P: a patch extracted from X; S: the window size of the extraction; $X_{i,j}$,$X_{u,v}$: the neighborhoods centered on pixel $x_{i,j}$ and $x_{u,v}$; $X_{f}$: the fusion matrix of $X_{i,j}$ and $X_{u,v}$; D: the dictionary of representation; A: the coefficient matrix.).

In order to further exploit the local spectral similarity through NLSS, this paper proposes a novel GSRC-based approach for HSI classification. As illustrated in Figure 1, the proposed method considers both the nonlocal spatial self-similarity by conducting a search of nonlocal patches and the local spectral similarity by exploration of the found patches. Specifically, the proposed method can be implemented in three steps: (1) Nonlocal search of spatial similar patches, where the most similar non-overlapped patch towards the patch containing the current testing pixel is located in the whole scene. (2) Local search of spectral similar pixel, which measures the most similar pixel in the spectral domain to the testing pixel in its found nonlocal patch in step (1). (3) Spectral-spatial structure-based representation, where the neighborhoods of the testing pixel and its similar pixel in step (2) are fused together to be processed by a GSRC architecture. The final classification result is determined by the class with minimum representation error. The main contribution of the proposed approach, denoted as NSLS-GSRC (nonlocal spatial and local spectral similarity based GSRC), can be summarized as follows:

1. Local spatial information is first considered in the proposed framework. Compared with the traditional SR-based methods, our approach represents the testing pixel with its neighborhood simultaneously, which allows for the consideration of local spatial consistency.
2. Nonlocal spatial information is then emphasized by our proposed method. As an important check of local spatial consistency, NLSS is integrated with local similarity to provide a global spatial constraint under local and nonlocal spatial consistency.
3. Local spectral information is further exploited through NLSS. Based on the exploration of local spectral similarity, our proposed NSLS-GSRC takes into account both the nonlocal spatial and the local spectral information, and contributes to a more comprehensive representation based on a group structured prior of GSRC.

The remainder of this paper is organized as follows: Section 2 formulates the representation models adopted in this paper. Section 3 details the proposed NSLS-GSRC approach. Section 4 evaluates the performances of our method compared with those of other related hyperspectral image classifiers, using data sets collected by the Airborne Visible/Infrared Imaging Spectrometer (AVIRIS) over the Indian Pines site in northwestern Indiana (USA), Salinas Valley in California USA), and the Reflective Optics Spectrographic Imaging System (ROSIS) over the University of Pavia in Italy. Section 5 presents some concluding remarks.

## 2. Background

Compared with the traditional processing techniques for HSI such as band fitting and support vector machine (SVM) [38,39,40], representation-based methods have gained great interest due to their no assumption of data density distribution. By representing testing pixels as a linear combination of a small subset of labeled pixels, they have been proven to provide rather plausible results. One of the classic models is SRC, which has been widely exploited for HSI. Later, joint SRC (JSRC) was developed to introduce local spatial correlation to the SRC framework. However, obstacles remain in the development of this method, such as the inherent instability of sparse coefficients [41]. Therefore, GSRC is designed to reconstruct the dictionary based on group structured prior, such that the representation can be processed in a more comprehensive model [28].

### 2.1. Sparse Representation-Based Classifier (SRC)

Given a hyperspectral image denoted as **X**, which contains a total of *B* spectral bands, *K* labeled classes and *N* pixels, where *N* = *r* × *c*, and *r* and *c* are the length of row and column of **X**. Let **x***_i,j_* define a testing pixel in **X** with the location (*i,j*). Traditional SRC represents the testing pixel **x***_i,j_* by a sparse linear combination of labeled samples under a *l*_1_-norm constraints of coefficients as follows:

(1) $$\min\frac{1}{2}{\left||x_{i,j}-D\alpha |\right|}_{2}^{2}+\lambda {\left||\alpha |\right|}_{1},$$

where **D** is defined as a dictionary composed of random selected labeled samples from each class, **α** is a weight vector corresponding to **D** towards the **x***_i,j_* during the representation, and *λ* is a regularization parameter. ${\left\|g\right\|}_{1}$ and ${\left\|g\right\|}_{2}$ denote the *l*_1_-norm and *l*_2_-norm constraints. The class label is determined by the minimum residual error between **x***_i,j_* and its approximation as follows:

(2) $$class(x_{i,j})=\arg\underset{k}{\min}{\left||x_{i,j}-D\delta_{k}(\alpha )|\right|}_{2}^{2},$$

where *k* ∈ {1,…,*K*} is the class index, and *δ_k_*(g) is an indicator operation which can zero out all the elements in **α** that does not belong to class *k*.

### 2.2. Joint SRC (JRSC)

SRC focuses on the spectral characteristics, and it largely neglects the spatial correlation of contextual information in a remote sensing scene. In HSI, pixels in a neighborhood usually consist of similar materials. This spatial prior can be incorporated with a representation process, so that the local spatial coherence can be exploited to avoid some misclassified errors caused by the phenomenon of the same material with different spectra. Therefore, the main principle of JSRC is to represent pixels in close proximity with a common sparsity support. Considering $X_{i,j}$ is a $B\times {\left(S\right)}^{2}$ sized matrix which is transformed by a S×S sized neighborhood center on $x_{i,j}$ from the original scene, the objective function of the JSRC is represented as follows:

(3) $$\underset{A}{\min}\frac{1}{2}{\left\|X_{i,j}-DA\right\|}_{F}^{2}+\lambda {\left\|A\right\|}_{2,1},$$

where A is a coefficient matrix composed of the weight vectors corresponding to pixels in $X_{i,j}$, and ${\left\|\cdot \right\|}_{F}$ is the Frobenius norm. Note that ${\left\|A\right\|}_{2,1}=\sum_{i=1}^{n}a^{i}$ is a $l_{2,1}$-norm constraint, n is the number of labeled samples selected in D, and $a^{i}$ is defined as the $i_{th}$ row of A, which is different from α (α is the column vector of A). The class label of the centered pixel $x_{i,j}$ is determined by the minimum residual error with the following function:

(4) $$class(x_{i,j})=\arg\underset{k}{\min}{\left\|X_{i,j}-D\delta_{k}(A)\right\|}_{2}^{2},$$

where $\delta_{k}(\cdot )$ is set to zero out all the elements in A that does not belong to class k.

### 2.3. Group SRC (GRSC)

In JSRC and SRC, the testing pixels are represented by individual atoms of a dictionary. The dictionary of representation-based classifiers has an inherent group structured property, which means that the atoms from the same class can be grouped together and the pixels can be represented by groups of atoms. By encouraging coefficients of only certain groups to be active and the remaining groups inactive, the representation can obtain a more centralized optimization response. Therefore, GSRC reconstructs the dictionary as $D=(D_{1},\ldots ,D_{K})$ with each column vector of $D_{k}$ representing a labeled sample randomly selected from class k. Group Lasso optimization can be exploited to sum up the $l_{2}$-norm of group coefficients based on a sparsity prior [42], such that the optimization function of GSRC can be represented as follows:

(5) $$\underset{A}{\min}\frac{1}{2}{\left\|X_{i,j}-DA\right\|}_{F}^{2}+\lambda \sum_{g\in G}\omega_{g}{\left\|A_{g}\right\|}_{2},$$

where $g\subset \{G_{1},\ldots ,G_{K}\}$ defines the K groups and $A_{g}$ represents the coefficient matrix of each group corresponding to the subdictionary $D_{k}$ in D, and $\omega_{g}$ is a regularization parameter which is adopted to compensate for different sizes of groups. $\sum_{g\in G}{\left\|A_{g}\right\|}_{2}$ can be regarded as a group-level $l_{2,1}$-norm constraint, and the GSRC can degenerate to a JSRC if the group size reduces to one.

## 3. Proposed Approach

As introduced in Section 2.3, GSRC exploits the prior structured group of a dictionary, and integrates the spatial information from the local area for better optimization. In order to satisfy the homogeneity assumption and the local spatial consistency during the representation, GSRC usually considers a small neighborhood around the testing pixel. Assuming that this small neighborhood contains some spectral abnormal pixels (caused by the presence of same material with different spectra), GSRC probably misclassifies the testing pixel. However, in this project, when we consider a larger patch which contains the current testing neighborhood, its local spectral similar area in a nonlocal spatial similar patch can be found in the original scene, such that this new area can strengthen the constraints towards the problem testing neighborhood during the representation, and obtain an improved response. Therefore, it is reasonable to exploit the nonlocal spatial and local spectral similarities of image for adequate supplement to the discrimination of materials of interest in spectral-spatial frameworks. 

As illustrated in Figure 1, let $P_{i,j}$ represent a $S_{1}\times S_{1}$ sized patch with the center pixel of $x_{i,j}$, the proposed NSLS-GSRC firstly searches the most spatially similar non-overlapped patch from $P_{a,b}$ towards $P_{i,j}$. Then, the most similar spectral pixel from $x_{u,v}$ towards $x_{i,j}$ is located in the found nonlocal patch $P_{a,b}$. After that, two $S_{2}\times S_{2}$ sized neighborhoods $X_{i,j}$ and $X_{u,v}$ centered on $x_{i,i}$ and $x_{u,v}$ can be extracted from the original scene X. With a fusion process, the new testing matrix can now be sparsely represented by GSRC. The final class label of $x_{i,j}$ is determined by the minimum representation error with group structured dictionary D and coefficient matrix A. The details of this process are presented in the following subsections.

### 3.1. Nonlocal Spatial and Local Spectral Similarity (NSLS)

In order to implement the search of a nonlocal patch towards each pixel in the whole image, X needs to be expanded to a $\left(r+S_{1})\times (c+S_{1}\right)$ sized image. Then, we start to extract $S_{1}\times S_{1}$ sized patches from the upper left corner of the expanded image with step of one pixel, such that patches centered on each pixel from the original scene can be obtained. For example, P denotes the set of all the patches, where $P_{i,j}\in P$ represents the patch centered on $x_{i,j}$. The first step of determination of our proposed NSLS is to find the most similar nonlocal spatial patch $P_{a,b}\in P$ towards $P_{i,j}$. This process adopts two principles: 1. $P_{a,b}$ should be the most similar patch-based one relative to $P_{i,j}$ in the search area. 2. $P_{a,b}$ and $P_{i,j}$ cannot overlap, i.e., either the distance between abscissa or ordinate of their centered pixels should be larger than the side length of the extracted patch. Therefore, the determination of $P_{a,b}$ can be represented as follows:

(6) $$\begin{array}{c} \underset{P_{a,b}}{\min}\;\underset{x^{m}\in P_{i,j},x^{n}\in P_{a,b}}{dist(P_{i,j},P_{a,b})}=\sum_{m,n=1}^{W_{1}{}^{2}}d(x^{m},x^{n})\\ s.t.(\left|i-a\right|>S_{1})\vee (\left|j-b\right|>S_{1})=1 \end{array},$$

where $x_{m}$ and $x_{n}$ are the $m_{th}$ and $n_{th}$ pixel vector in $P_{i,j}$ and $P_{a,b}$, and $d(x_{m},x_{n})$ calculates the Euclidian distance between them. (a,b) is the coordinate of centered pixel $x_{a,b}$ of $P_{a,b}$, and the constraint condition is corresponding to the second principle listed above. After $P_{a,b}$ is obtained, the most similar spectral pixel $x_{u,v}$ compared with $x_{i,j}$ can be located in $P_{a,b}$ as follows:

(7) $$\underset{x_{u,v}}{\min}\;\underset{x_{u,v}\in P_{a,b}}{dist(x_{i,j},x_{u,v})}=\sum_{b=1}^{B}d(x_{i,j}^{b},x_{u,v}^{b}),$$

where $x_{i,j}^{b}$ and $x_{u,v}^{b}$ are the values of $x_{i,j}$ and $x_{u,v}$ in $b_{th}$ band, and $d(x_{i,j}^{b},x_{u,v}^{b})$ calculates their Euclidian distance. Since the measurement between $P_{i,j}$ and $P_{a,b}$ is on a patch-based level, $x_{u,v}$ obtained here might not be located at the center of $P_{a,b}$ (as illustrated in Figure 1), i.e., $x_{u,v}$ and $x_{a,b}$ are not the same pixel. Therefore, compared with other related NLSS-based methods, the innovation of the proposed NSLS-GSRC approach is the further exploration of local spectral similarity through nonlocal spatial similarity [32,33].

### 3.2. NSLS-GSRC

Following the formulation described in previous sections, $x_{u,v}$ is found and can be considered the most similar local spectral pixel in nonlocal spatial similar patch $P_{a,b}$ towards the current testing pixel $x_{i,j}$ in its neighboring patch $P_{i,j}$. Then, a $S_{2}\times S_{2}$ sized neighborhood centered on $x_{u,v}$ can be obtained and transformed to a $B\times {\left(S_{2}\right)}^{2}$ sized matrix denoted as $X_{u,v}$. After that, $X_{u,v}$ is fused with $X_{i,j}$ by a pixel-by-pixel average process. The fusion result can be then processed in the representation-based framework by Group Lasso optimization. Finally, the objective function of the proposed NSLS-GSRC method can be represented as follows:

(8) $$\underset{A}{\min}\frac{1}{2}\left\|X_{f}-DA\right\|+\lambda \sum_{g\in G}\omega_{g}{\left\|A_{g}\right\|}_{2},$$

where $X_{f}=\{\sum_{j=1}^{S_{2}^{2}}(x^{j}+x^{'}{}^{j})|x^{j}\in X_{i,j},x^{'}{}^{j}\in X_{u,v}\}$ denotes the fusion result of $X_{i,j}$ and $X_{u,v}$. The final class label of testing pixel $x_{i,j}$ is determined by the minimum total residual error as follows:

(9) $$class(x_{i,j})=\underset{k}{\arg\min}{\left\|X_{f}-D\delta_{k}(A)\right\|}_{F}^{2}.$$

where $\delta_{k}(A)$ represents the operation to zero our all the elements in A that do not belong to class k. The pseudo code for the proposed NSLS-GSRC method is shown in Algorithm 1.


**Algorithm 1. The proposed NSLS-GSRC method**
**Input:** A HSI image X, dictionary D is constructed by class orders and randomly selected from the labeled samples, a testing pixel $x_{i,j}$ and the patch-size parameters $S_{1}$ and $S_{2}$
*Step 1*: Extract a $S_{1}\times S_{1}$ sized patch denoted as $P_{i,j}$ with centered pixel of $x_{i}{}_{,j}$ from X;*Step 2*: Search the nonlocal spatial similar patch $P_{a,b}$ towards $P_{i,j}$ according to Equation (6);*Step 3*: Search the local spectral similar pixel $x_{u,v}$ in $P_{a,b}$ towards the current testing pixel $x_{i,j}$ according to Equation (7);*Step 4*: Extract two $S_{2}\times S_{2}$ sized patches with the center pixels of $x_{i,j}$ and $x_{u,v}$, and transform them in to two-dimensional formed matrix denoted as $X_{i,j}$ and $X_{u,v}$;*Step 5*: Obtain the fusion matrix $X_{f}$ of $X_{i,j}$ and $X_{u,v}$, and use them using GSRC to obtain the coefficient matrix A according to Equation (8);*Step 6*: Compute the minimun total residual error and identify the class label of the testing pxiel $x_{i,j}$ according to Equation (9);**Output:** class ($x_{i,j}$).

## 4. Experimental Results

In this section, the proposed NSLS-GSRC method is evaluated using three widely used hyperspectral data sets. The first one is the Indian Pines scene collected by the Airborne Visible/Infrared Imaging Spectrometer (AVIRIS) (NASA Jet Propulsion Laboratory, Los Angeles, CA, USA), with spectral coverage ranging from 0.25 to 2.4 μm and geometric resolution of 20 m per pixel. The scene contains 145×145 pixels, with 220 spectral bands. The ground reference contains sixteen classes, which are associated with different kinds of crops. The second scene is the Salinas scene recorded by the AVIRIS sensor. The spatial resolution of this image is 3.7 m per pixel. The scene contains 512×217 pixels, with 224 spectral bands ranging from 0.9 to 1.4 μm. The ground reference contains sixteen ground-truth classes. The last scene is University of Pavia scene captured by the Reflective Optics Spectrographic Imaging System (ROSIS) (DLR Institute of Optoelectronics, Berlin, Germany), with spectral coverage from 0.43 to 0.86 μm and geometric resolution of 1.3 m per pixel. The scene consists of 610×340 pixels, with 103 spectral bands and nine ground-truth classes. For comparative purposes, several competing spectral and spectral-spatial classifiers are considered in experiments, such as SVM [43], SVM based Markov Random Field (SVM-MRF) [44], SRC [16], CRC [17], JSRC [20], GSRC [29]. In addition, NL-means based SRC (NL-SRC) is also carried out for comparison similar to the literature [35] to evaluate the proposed NSLS-GSRC method. We conduct 20 Monte Carlo runs while varying the randomly selected labeled samples, and report the corresponding results.

### 4.1. Parameter Settings

**Figure 2:**
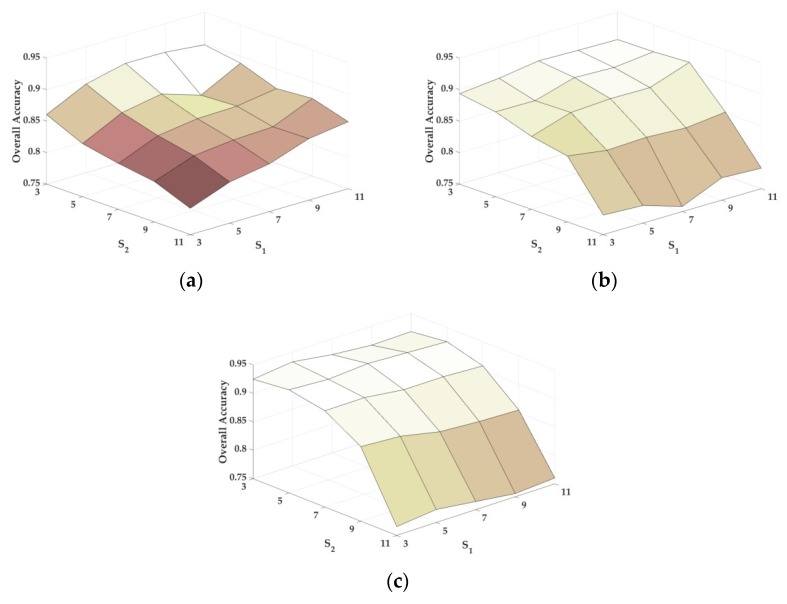
Parameter tuning (e.g. $S_{1}$ and $S_{2}$ ) of the proposed NSLS-GSRC method: (**a**) AVIRIS Indian Pines scene; (**b**) AVIRIS Salinas scene; (**c**) ROSIS University of Pavia scene.

In the experiments of this paper, the regularization parameter *λ* for the representation-based methods ranges from 10^−3^ to 10^−1^, *ω_g_* for the group sparse representation-based methods is set to 1 due to the same number of labeled samples randomly selected per class in experiments. The parameters of the other methods have been optimized by means of a fivefold cross-validation according to the procedure provided in the literature [16,17,20,29,43,44]. In particular, the values of *S*_1_ and *S*_2_ are tested extensively and illustrated in Figure 2.

As shown in Figure 2, with a fixed number of labeled samples, the overall classification accuracy is relatively stable with respect to $S_{1}$, but obviously decreases with respect to $S_{2}$. This is because $S_{2}$ defines the size of neighborhood in group sparse representation, and it needs to be constrained in a relatively small range such that to satisfy the homogeneity assumption and the local spatial consistency. Instead, $S_{1}$ decides on the searching patch-size which determines the nonlocal spatial and local spectral similarity, and it is allowed to range in a relatively larger range compared with $S_{2}$ due to the global structure priors. According to the results of Figure 2, we adopt $S_{1}=7$ and $S_{2}=3$ for the Indian Pines scene, as well as $S_{1}=11$ and $S_{2}=7$ for the Salinas scene. Finally, for the University of Pavia scene, $S_{1}$ and $S_{2}$ are chosen to be 5 and 3.

### 4.2. Experiments with the AVIRIS Indian Pines Scene

**Figure 3:**
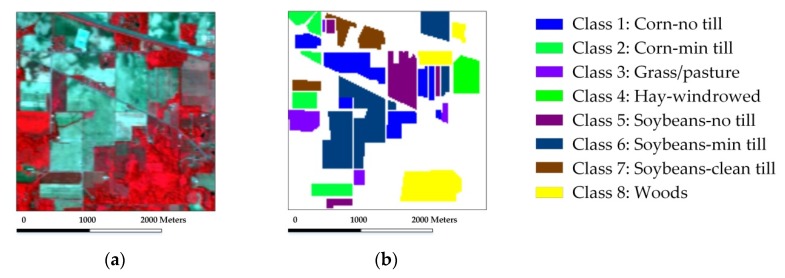
The AVIRIS Indian Pines scene: (**a**) false-color composite image; (**b**) reference map.

**Figure 4:**
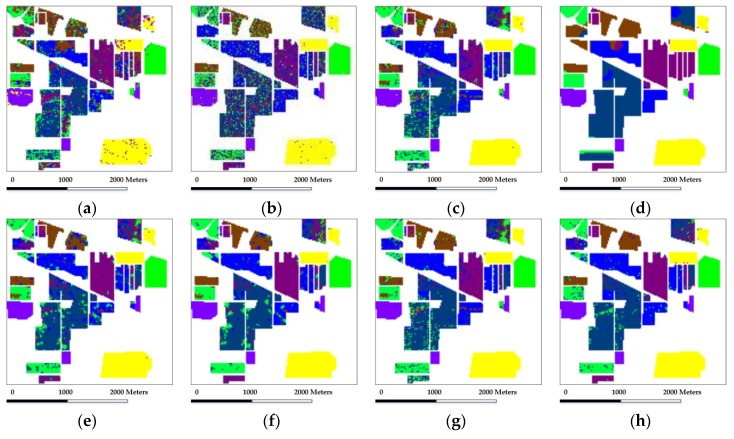
Classification maps obtained by the different tested method for the AVIRIS Indian Pines scene (OA are in parentheses): (**a**) SVM(63.57%); (**b**) CRC (70.89%); (**c**) SRC (78.18%); (**d**) SVM-MRF (84.31%); (**e**) JSRC (81.47%); (**f**) GSRC (86.84%); (**g**) NL-SRC (85.04%); (**h**) NSLS-GSRC (90.54%).

In the experiment with the Indian Pines scene, eight mutually exclusive classes with a total of 8624 labeled samples are adopted from the reference data to avoid some classes with very small training samples, and also to satisfy the sparsity requirement in the process of representation. Figure 3 shows the false-color composite of the image and the reference map. Our first test randomly selects 50 labeled samples per class with a total of 400 samples (which represents approximately 4.6% of the labeled samples) for training and dictionary construction, where the remaining samples are used for validation. Table 1 shows the overall and individual classification results of different testing methods. Figure 4 shows the classification maps obtained by the different testing methods. Several conclusions can be drawn.

1. Compared with SVM, both SRC and CRC provide considerable individual classification accuracies and a slightly better overall classification result. Firstly, it indicates that the representation-based methods can indeed provide plausible results towards traditional models for HSI classification. Also, it is a basis support for the framework of sparse representation exploited in our proposed method.
2. Compared with SVM and SRC, SVM-MRF and JSRC achieve higher overall classification accuracies and provide more homogeneous classification maps, demonstrating that the incorporation of local spatial information can bring improvement to the classifiers in the spectral domain. The improvement supports the homogeneity assumption and the local spatial consistency in the spectral-spatial framework for the classification of HSI.
3. Compared with SVM-MRF and JSRC, GSRC achieves better classification results which proves that group structured priors contribute to a more comprehensive integration of spectral and local spatial information. The improvements of GSRC over JSRC indicate the superiority of group sparsity framework and the Group Lasso optimization.
4. Compared with SRC, NL-SRC brings improvement in classification result that is similar to GSRC. On one hand, it proves that the incorporation of nonlocal spatial information is effective for the classifiers in the spectral domain. On the other hand, it also indicates that both the nonlocal spatial similarity and the local spatial consistency improve the sparse representation framework for the classification of HSI.
5. The proposed NSLS-GSRC outperforms GSRC and NL-SRC, which firstly demonstrates that the integration of both nonlocal and local spatial information contributes to a more comprehensive consideration of structured priors compared with either of them. In addition, it also indicates that the exploration of local spectral similarity through nonlocal spatial similarity provides more effective means for the discrimination of materials in spectral-spatial frameworks. Furthermore, it proves that the combination of global structured priors and group structured priors, i.e., NSLS and GSRC in our proposed method, can bring significant improvement for the classification of HSI.

In general for this case, the proposed NSLS-GSRC obtains an overall accuracy of 90.54%, which is 5.5% and 3.7% higher than NL-SRC and GSRC, and also 12.36% higher than SRC, respectively. For individual class accuracy, it also provides considerable results, especially for classes 1 and 5. The classification maps in Figure 4 confirm the improvement achieved by the proposed method.

**Figure 5:**
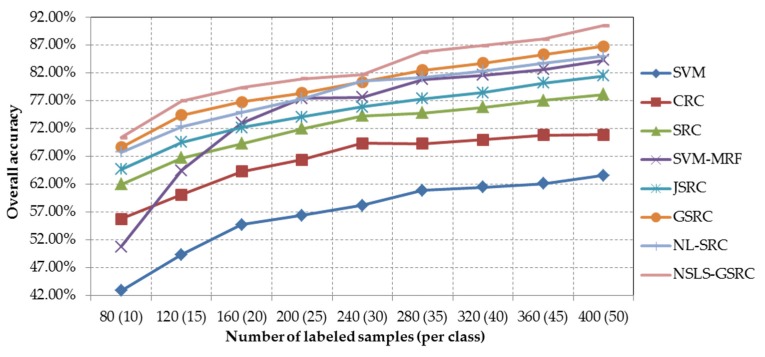
Overall classification accuracy obtained by the different tested methods versus different number of labeled samples for the AVIRIS Indian Pines scene.

In the second test with the Indian Pines scene, the proposed NSLS-GSRC is compared with other test methods using a different number of labeled samples (from 10 to 50 samples per class). Figure 5 and Table 2 show the overall classification accuracies obtained by the different testing methods, as a function of the number of labeled samples used. As shown by Figure 5 and Table 2, several conclusions can be drawn:

1. Overall classification accuracies are generally positively correlated with the number of labeled samples selected for training and dictionary construction. The improvement is relatively obvious with the situation where the method has a limited number of labeled samples, which can be seen from the variation trend of overall classification accuracies in Figure 5.
2. The integration of spatial information contributes to more accurate discrimination of materials. In particular, both local spatial information and nonlocal spatial information both contribute to better characterizing the image in the spectral-spatial domain, which can be seen from the improvement of overall classification accuracies of SVM-MRF, JSRC, GSRC and NL-SRC when compared with the original counterparts, i.e., SVM and SRC.
3. The combination of local and nonlocal spatial information contributes to more comprehensive consideration of global structured priors. This is especially true for the proposed method, because with a further exploration of local spectral similarity through nonlocal spatial similarity, NSLS-GSRC brings reliable and stable improvement of classification in comparison with other methods either using local or nonlocal spatial information only.

### 4.3. Experiments with the AVIRIS Salinas Scene

**Figure 6:**
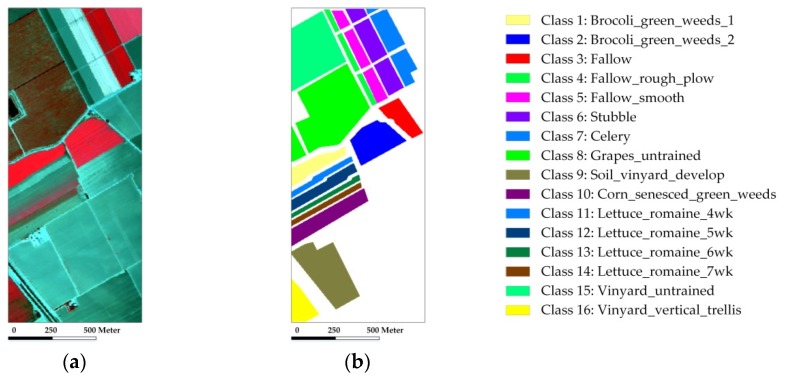
The AVIRIS Salinas scene: (**a**) false-color composite image; (**b**) reference map.

**Figure 7:**
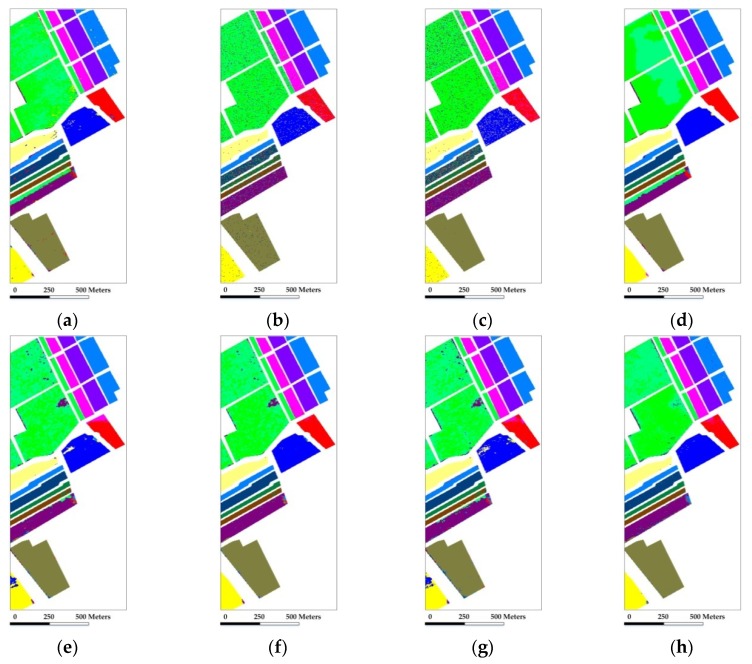
Classification maps obtained by the different tested method for the AVIRIS Salinas scene (OA are in parentheses): (**a**) SVM(81.63%); (**b**) CRC (81.03%); (**c**) SRC (81.49%); (**d**) SVM-MRF (85.27%); (**e**) JSRC (84.49%); (**f**) GSRC (88.11%); (**g**) NL-SRC (88.62%); (**h**) NSLS-GSRC (91.06%).

The Salinas scene used in our second experiment was recorded by the AVIRIS sensor over the Salinas Valley, California. Figure 6 shows the false-color composite of the image and the reference map which contains a total of 54,129 labeled samples. We first randomly select 20 labeled samples per class with a total of 320 samples (which represents approximately 0.6% of the labeled samples) for training, where the remaining samples are used for testing. The classification results and maps obtained by different comparison methods are provided in Table 3 and Figure 7.

As shown in Figure 7 and Table 3, SRC and CRC provide comparable results with SVM in the spectral domain. In the spatial domain, GSRC, JSRC and SVM-MRF bring significant improvement relative to SRC and SVM by integrating local spatial consistency. On the other hand, NL-SRC also achieves an obvious increase of classification accuracy relative to SRC with consideration of nonlocal spatial self-similarity. Last but not least, the proposed NSLS-GSRC obtains the best classification result with an overall accuracy of 91.06%, which is 2.44% and 2.95% higher than NL-SRC and GSRC, also 9.57% higher than SRC, respectively. In addition, it brings considerable improvements for individual class accuracy, especially for class 14 and 15, which can be observed from the classification map illustrated in Figure 7.

**Figure 8:**
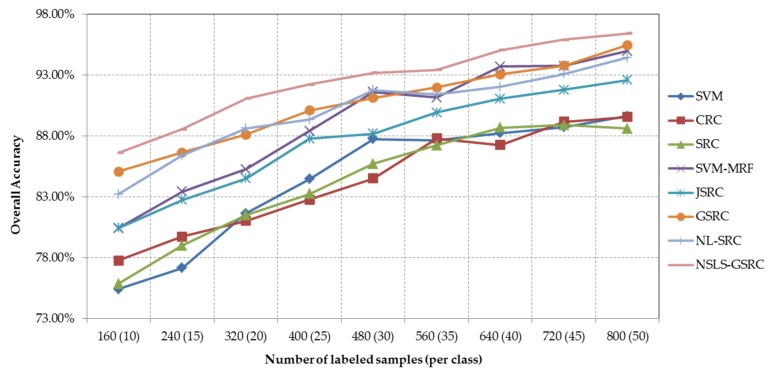
Overall classification accuracy obtained by the different tested methods versus different number of labeled samples for the AVIRIS Indian Pines scene.

Our second test of the Salinas scene evaluates the proposed NSLS-GSRC method with a varying size of labeled samples (from 10 to 50 samples per class). Figure 8 and Table 4 show the overall classification accuracies obtained by different testing methods, as a function of the number of labeled samples adopted for training and dictionary construction. As shown in Table 4 and Figure 8, the improvement of JSRC and GSRC relative to SRC proves the effectiveness of integration of local spatial information with consideration of spatial coherence. The effectiveness of nonlocal spatial information is demonstrated by the increase of overall accuracies acquired by NL-SRC in comparison with SRC. The superiority of local spectral similarity through NSLS is confirmed by the best overall classification accuracies obtained by the proposed NSLS-GSRC method in all cases, which allows for an overall consideration of local and nonlocal spatial information.

### 4.4. Experiments with the ROSIS University of Pavia Scene

**Figure 9:**
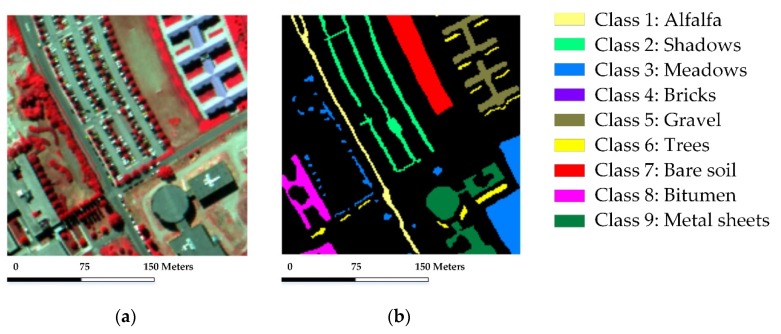
The ROSIS University of Pavia scene: (**a**) false-color composite image; (**b**) reference map.

In the experiment with the ROISIS University of Pavia scene, a 180×180 pixel-size patch with a total of 7398 labeled samples from nine classes is extracted from the original scene with consideration of time efficiency. Figure 9 shows the false-color composite image of the extracted region and the corresponding reference map.

**Figure 10:**
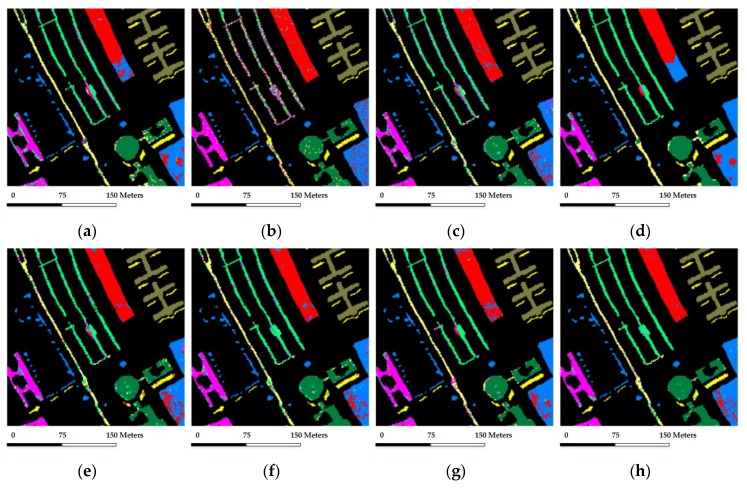
Classification maps obtained by the different tested method for the University of Pavia scene (OA are in parentheses): (**a**) SVM (86.73%); (**b**) CRC (79.17%); (**c**) SRC (86.58%); (**d**) SVM-MRF (90.33%); (**e**) JSRC (88.54%); (**f**) GSRC (90.71%); (**g**) NL-SRC (91.31%); (**h**) NSLS-GSRC (93.27%).

Our first test randomly selected 30 labeled samples per class with a total of 270 samples for training and dictionary construction (which represents approximately 3.6% of the labeled samples), while the remaining samples are used for validation. Table 5 reports the overall and class-specific accuracies of different testing methods, where Figure 10 shows the corresponding classification maps for this case.

As shown in Figure 10 and Table 5, SVM provides considerable results in similar with SRC and CRC in the spectral domain. The methods based on local spatial consistency and structured priors generally bring improvement to their original counterparts, for instance, GSRC outperforms SRC. The methods based on nonlocal spatial similarity offer support for the notion of better consideration of global structure priors, for instance, NL-SRC outperforms SRC. Furthermore, the proposed method brings better characterization of spatial and spectral information based on nonlocal spatial and local spectral similarity, and achieves a more comprehensive discrimination of materials. In general for this case, the proposed NSLS-GSRC obtains an overall accuracy of 93.27%, which is 2.96% and 2.56% higher than NL-SRC and GSRC, also 6.69% higher than SRC, respectively. In addition, the proposed NSLS-GSRC provides reliable individual classification accuracy for each class, especially for classes 2, 7 and 8. It can also be seen from the more homogenous details of the classification map obtained by NSLS-GSRC in Figure 10, which confirms the improvement.

**Figure 11:**
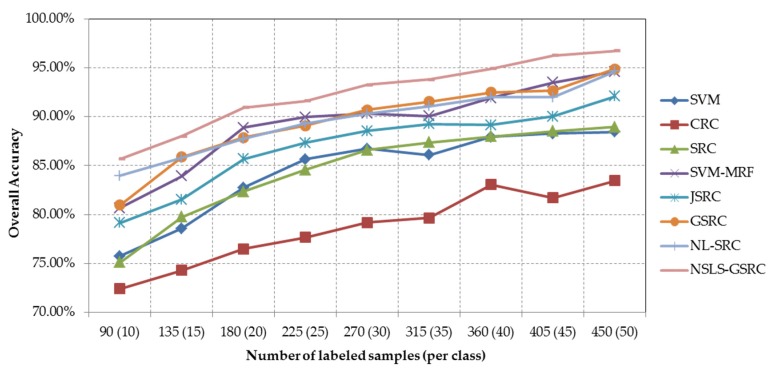
Overall classification accuracy obtained by the different tested methods versus different number of labeled samples for the ROSIS University of Pavia scene.

Our second test of the University of Pavia scene evaluates the proposed NSLS-GSRC with a varying size of labeled samples (from 10 to 50 samples per class). Figure 11 and Table 6 show the overall classification accuracies obtained by different methods tested, as a function of the number of labeled samples used for training and dictionary construction. Though CRC obtains relatively poor results in this case, SRC provides competitive overall classification accuracies towards SVM with the increase of the number of training samples in the spectral domain. In the spatial domain, stable increases are obtained with consideration of local spatial consistency, which can be concluded from the comparisons and their trend of SVM-MRF with SVM, and JSRC/GSRC with SRC. On the other hand, improvement are also brought by the combination of nonlocal spatial self-similarity and spectral-domain SR, which is indicated by the performance of NL-SRC versus GSRC, especially for the situation of the limited training samples. In the spectral-spatial domain for all cases, the proposed NSLS-GSRC method achieves the best results in comparison with the other related methods, which demonstrates that the integration of nonlocal spatial and local spectral information achieves more comprehensive discrimination of materials.

Synthesizing the results and analysis in above three experiments, the proposed NSLS-GSRC method obtains comprehensive and considerable overall and individual classification accuracies, and provides more homogenous details in classification maps compared with other related methods. The classification results with varying number of training samples further verifiy the stability of the proposed method. In general, we would like to emphasize that the proposed NSLS-GSRC based on the integration of nonlocal spatial and local spectral similarity is reliable and stable for the classification of HSI.

## 5. Conclusions

Hyperspectral images are characterized by their abundant spectral and spatial information. Considering the high redundancy and correlation among spectral bands, it has been demonstrated that the inherent sparse property can be exploited for more accurately discriminating materials under examination. In this context, SR-based methods have shown their effectiveness by representing pixel with a linear combination of labeled samples, and obtained a certain success in spectral domain. In order to better characterize the image for classification, researches have been focused on two major aspects to incorporate the spatial information of image. In the local spatial domain, JSRC and GSRC have been designed to simultaneously represent the pixel in neighborhood based on the local spatial coherence. In the nonlocal spatial domain, NLSS is presented to measure the similarity of pixels based on the nonlocal structured priors. With a further investigation of more comprehensive classification, a new framework should allow the exploration of spectral similarity through NLSS and combination of nonlocal and local spatial information in the spectral-spatial domain.

In this paper, we proposed a new classification framework to exploit nonlocal spatial and local spectral similarity based on group sparse representation for hyperspectral image. The main contribution of the proposed method, abbreviated as NSLS-GSRC, includes the further exploitation of spectral similarity through nonlocal spatial self-similarity, and its incorporation with group structure-based sparse representation based on local spatial consistency. Experiments based on three real hyperspectral data sets demonstrate that the proposed NSLS-GSRC outperforms other related methods for the classification performance.

## Figures and Tables

**Table 1:** Classification accuracies (in percent) obtained by the different tested methods for the AVIRIS Indian Pines scene. In all cases, 400 labeled samples in total (50 samples per class) were used for training. The best results are in bold.

Class	Samples	SVM	CRC	SRC	SVM-MRF	JSRC	GSRC	NL-SRC	NSLS-GSRC
1	1460	49.96%	76.22%	75.62%	68.85%	69.18%	79.18%	85.75%	**86.64%**
2	834	50.13%	60.35%	80.10%	70.59%	82.85%	**89.33%**	87.41%	88.25%
3	497	86.87%	94.18%	97.38%	95.98%	96.78%	**98.39%**	97.99%	**98.39%**
4	489	98.51%	97.80%	**100.00%**	99.53%	**100.00%**	**100.00%**	**100.00%**	**100.00%**
5	968	62.57%	71.14%	69.52%	84.67%	89.15%	93.18%	76.24%	**96.69%**
6	2468	51.67%	45.73%	62.03%	**82.21%**	69.29%	75.89%	72.37%	81.93%
7	614	64.28%	78.46%	81.76%	**98.17%**	80.29%	88.76%	91.21%	95.28%
8	1294	90.29%	96.76%	99.77%	98.46%	99.61%	99.69%	**99.92%**	99.38%
OA		63.57%	70.89%	78.18%	84.31%	81.47%	86.84%	85.04%	**90.54%**

**Table 2:** Overall accuracies (in percent) obtained by the different tested methods using different number of labeled samples (per class) for the Indian Pines scene. The best results are in bold.

Samples	SVM	CRC	SRC	SVM-MRF	JSRC	GSRC	NL-SRC	NSLS-GSRC
80 (10)	42.88%	55.76%	61.98%	50.74%	64.68%	68.68%	67.76%	**70.41%**
120 (15)	49.34%	60.15%	66.72%	64.50%	69.58%	74.42%	72.40%	**76.97%**
160 (20)	54.73%	64.29%	69.27%	73.01%	72.17%	76.82%	74.97%	**79.40%**
200 (25)	56.39%	66.42%	71.97%	77.47%	74.13%	78.41%	77.35%	**80.97%**
240 (30)	58.20%	69.34%	74.33%	77.63%	75.89%	80.39%	80.50%	**81.73%**
280 (35)	60.87%	69.30%	74.83%	80.84%	77.35%	82.51%	81.23%	**85.76%**
320 (40)	61.46%	70.02%	75.82%	81.64%	78.47%	83.82%	82.39%	**86.97%**
360 (45)	62.08%	70.85%	77.09%	82.67%	80.19%	85.31%	83.82%	**88.12%**
400 (50)	63.57%	70.89%	78.18%	84.31%	81.47%	86.84%	85.04%	**90.54%**

**Table 3:** Classification accuracies (in percent) obtained by the different tested methods for the AVIRIS Salinas scene. In all cases, 320 labeled samples in total (20 samples per class) were used for training. The best results are in bold.

Class	Samples	SVM	CRC	SRC	SVM-MRF	JSRC	GSRC	NL-SRC	NSLS-GSRC
1	2009	97.35%	**99.50%**	99.40%	99.30%	**99.55%**	**99.95%**	99.90%	99.90%
2	3726	96.81%	95.73%	90.61%	99.36%	95.52%	99.06%	94.18%	**99.76%**
3	1976	94.84%	80.67%	82.39%	**98.29%**	86.54%	98.28%	90.59%	94.64%
4	1394	98.77%	85.08%	89.10%	98.95%	98.92%	**99.43%**	99.00%	98.28%
5	2678	95.25%	94.32%	92.16%	96.62%	99.22%	98.21%	98.43%	**99.44%**
6	3959	97.09%	99.77%	99.82%	98.33%	99.97%	**100.00%**	99.60%	99.90%
7	3579	97.84%	99.69%	99.75%	99.11%	99.83%	**99.94%**	99.22%	98.99%
8	11271	57.69%	62.36%	**84.43%**	69.02%	54.01%	65.39%	51.42%	76.51%
9	6203	94.68%	97.15%	**99.90%**	97.51%	98.28%	99.02%	96.34%	99.56%
10	3278	77.21%	83.89%	86.79%	83.89%	87.83%	**94.45%**	85.17%	87.16%
11	1068	90.88%	95.88%	97.19%	96.01%	99.44%	**99.81%**	97.38%	99.34%
12	1927	97.74%	63.00%	46.13%	**100.00%**	**100.00%**	**100.00%**	**100.00%**	99.22%
13	916	95.33%	63.21%	74.89%	96.94%	**99.78%**	99.02%	99.13%	99.24%
14	1070	91.75%	80.56%	83.08%	94.81%	93.08%	97.20%	92.15%	**97.57%**
15	7268	57.33%	50.66%	29.25%	54.28%	73.36%	71.08%	74.45%	**79.83%**
16	1807	94.40%	97.62%	93.47%	96.44%	88.93%	98.73%	87.88%	**98.78%**
OA		81.63%	81.03%	81.49%	85.27%	84.49%	88.11%	88.62%	**91.06%**

**Table 4:** Overall accuracies (in percent) obtained by the different tested methods using different number of labeled samples (per class) for the Salinas scene. The best results are in bold.

Samples	SVM	CRC	SRC	SVM-MRF	JSRC	GSRC	NL-SRC	NSLS-GSRC
160 (10)	75.42%	77.76%	75.85%	80.41%	80.44%	85.07%	83.21%	**86.62%**
240 (15)	77.16%	79.72%	78.98%	83.42%	82.74%	86.64%	86.39%	**88.54%**
320 (20)	81.63%	81.03%	81.49%	85.27%	84.49%	88.11%	88.62%	**91.06%**
400 (25)	84.45%	82.77%	83.22%	88.40%	87.77%	90.08%	89.33%	**92.22%**
480 (30)	87.73%	84.50%	85.70%	91.61%	88.19%	91.13%	91.77%	**93.17%**
560 (35)	87.65%	87.80%	87.23%	91.15%	89.93%	91.97%	91.42%	**93.43%**
640 (40)	88.23%	87.24%	88.68%	93.69%	91.04%	93.06%	92.02%	**95.05%**
720 (45)	88.71%	89.16%	88.90%	93.74%	91.79%	93.77%	93.08%	**95.89%**
800 (50)	89.63%	89.56%	88.59%	94.97%	92.59%	95.45%	94.43%	**96.41%**

**Table 5:** Classification accuracies (in percent) obtained by the different tested methods for the ROSIS University of Pavia scene. In all cases, 270 labeled samples in total (30 samples per class) were used for training. The best results are in bold.

Class	Samples	SVM	CRC	SRC	SVM-MRF	JSRC	GSRC	NL-SRC	NSLS-GSRC
1	526	83.33%	37.26%	65.78%	93.41%	68.82%	74.14%	**89.13%**	82.89%
2	1231	80.02%	95.69%	93.50%	74.53%	96.51%	94.88%	94.49%	**98.21%**
3	715	77.30%	**94.55%**	84.62%	89.65%	89.23%	91.47%	85.98%	93.01%
4	324	**100.00%**	**100.00%**	99.69%	**100.00%**	99.69%	99.38%	99.18%	**100.00%**
5	868	99.53%	99.77%	99.77%	99.65%	99.88%	**100.00%**	99.58%	**100.00%**
6	1140	86.73%	81.23%	83.07%	**91.24%**	78.86%	84.47%	89.50%	86.16%
7	1139	88.84%	94.91%	89.73%	91.32%	91.75%	92.54%	92.55%	**96.84%**
8	1095	81.66%	23.84%	72.79%	91.89%	82.92%	87.85%	86.99%	**92.69%**
9	360	**99.71%**	96.94%	96.67%	99.71%	88.89%	91.67%	99.46%	84.50%
OA		86.73%	79.17%	86.58%	90.33%	88.54%	90.71%	90.31%	**93.27%**

**Table 6:** Overall accuracies (in percent) obtained by the different tested methods using different number of labeled samples (per class) for the University of Pavia scene. The best results are in bold.

Samples	SVM	CRC	SRC	SVM-MRF	JSRC	GSRC	NL-SRC	NSLS-GSRC
90 (10)	75.75%	72.41%	75.11%	80.67%	79.16%	80.97%	83.96%	**85.67%**
135 (15)	78.59%	74.30%	79.74%	83.92%	81.53%	85.89%	85.79%	**88.00%**
180 (20)	82.76%	76.49%	82.34%	88.89%	85.67%	87.88%	87.75%	**90.93%**
225 (25)	85.65%	77.67%	84.58%	89.97%	87.33%	89.08%	89.31%	**91.61%**
270 (30)	86.73%	79.17%	86.58%	90.33%	88.54%	90.71%	90.31%	**93.27%**
315 (35)	86.08%	79.66%	87.37%	90.05%	89.25%	91.54%	91.08%	**93.81%**
360 (40)	87.93%	83.04%	87.99%	91.93%	89.16%	92.48%	91.97%	**94.93%**
405 (45)	88.27%	81.70%	88.53%	93.51%	90.04%	92.65%	91.99%	**96.27%**
450 (50)	88.44%	83.46%	88.94%	94.62%	92.08%	94.88%	94.63%	**96.73%**

## Abbreviations

The following abbreviations are used in this manuscript.

HSI	Hyperspectral Image
SR	Sparse Representation
JSR	Joint Sparse Representation
GSR	Group Sparse Representation
SRC	SR-based Classifier
CRC	Collaborative Representation-based Classifier
JSRC	JSR-based Classifier
GSRC	GSR-based Classifier
NL-means	Nonlocal means
NLSS	Nonlocal Self-Similarity
NSLS	Nonlocal Spatial and Local Spectral similarity
NSLS-GSRC	NSLS-based GSRC
SVM	Support Vector Machine
NL-SRC	NL-means-based SRC
AVIRIS	Airborne Visible/Infrared Imaging Spectrometer
ROSIS	Reflective Optics Spectrographic Imaging System
OA	Overall Accuracy
